# Metamaterials Application in Sensing

**DOI:** 10.3390/s120302742

**Published:** 2012-02-29

**Authors:** Tao Chen, Suyan Li, Hui Sun

**Affiliations:** 1 Mechanical & Power Engineering College, Harbin University of Science and Technology, Harbin 150080, China; 2 Center for Engineering Training and Basic Experimentation, Heilongjiang Institute of Science and Technology, Harbin 150027, China; E-Mail: lisuyan010@sina.com

**Keywords:** metamaterial, sensing, biosensor, thin-film sensor, strain sensor

## Abstract

Metamaterials are artificial media structured on a size scale smaller than wavelength of external stimuli, and they can exhibit a strong localization and enhancement of fields, which may provide novel tools to significantly enhance the sensitivity and resolution of sensors, and open new degrees of freedom in sensing design aspect. This paper mainly presents the recent progress concerning metamaterials-based sensing, and detailedly reviews the principle, detecting process and sensitivity of three distinct types of sensors based on metamaterials, as well as their challenges and prospects. Moreover, the design guidelines for each sensor and its performance are compared and summarized.

## 1. Introduction

Metamaterials are artificially made electromagnetic materials consisting of periodically arranged metallic elements which are less than wavelength of incident electromagnetic (EM) wave in size. Moreover, the materials can manipulate electromagnetic wave beams in surprising ways and exhibit some exotic electromagnetic properties which are not readily available in Nature, such as backward propagation, reverse Dollper effect, reverse Vavilov-Cerenkov effect [1], negative refraction [2,3], diffraction-limit breaking imaging [4–7], cloaking [8–10], *etc*. These exotic properties strongly depend on the geometry of metamaterial molecules rather than their composition [11]. Since they were theoretically proposed by Pendry *et al*. [2] and experimentally demonstrated by Smith *et al.* [3], metamaterials have attracted intensive research interest from microwave engineers and physicists in recent years because of their wide applications in super-lenses [4,12], slow light [13–15], data storage [16], optical switching [17] and so on. So far, researches into fabrication, design, and application of metamaterials have been extended to a fairly wide range of the EM spectrum including far-, mid-, and near-infrared regimes and even optical frequencies [18–20].

In addition, metamaterials can exhibit a strong localization and enhancement of fields so that they can be used to actually improve the sensor selectivity of detecting nonlinear substances and to enable detection of extremely small amounts of analytes [21]. Based on this property, many new or improved applications of metamaterials have been proposed recently. For example, using metamaterials instead of metal parts in surface plasmon resonance sensors was proposed to enhance the sensing performance [22], and utilizing metamaterials as high frequency sensors was also considered [23]. He *et al.* [24] studied resonant modes of a 2D subwavelength resonator, and the results showed it was suitable for biosensing. Alù *et al*. [25] proposed a method of dielectric sensing by using ε near-zero narrow waveguide channels. Shreiber *et al.* [26] developed a novel microwave nondestructive evaluation sensor by using metamaterial lens to detect material defects even as small as a wavelength. Zheludev [27] analysed the future trends of metamaterials, and pointed out that sensor applications are another growing area of metamaterial researches. Huang *et al.* [28] studied the performance of metamaterial sensors, and the results showed that the sensitivity and resolution of the sensors can be greatly enhanced by metamaterials. These open new degrees of freedom in sensor design, which promises a sensitivity boost and a facilitated readout.

Currently, the metamaterial science has reached a high degree of sophistication. As a result of continuous progress in design and fabrication of metamaterials on the submicron and nanometer scales, novel enhanced properties of metamaterials can be created. Thus, the interdisciplinary boundary between metamaterials science and sensing technology has become a fertile ground for new scientific and technological development. Moreover, the considerable advances have been made on research into sensors based on metamaterials, and they have led to the development of various metamaterial-based components to detect the information of substance and circumstance.

It is well known that sensing devices can detect a small change, depending on the following four criteria: first, the sensors must have an operating frequency low enough to avoid the background and substrate absorption. This poses a significant challenge as conventional sensing devices have a limited area, and such reduced space tends to increase the operating frequency of sensors. Therefore, it is required to maintain a small layout of the sensor while decreasing its operating frequency as much as possible. Second, the sensors must produce a strong and measurable readout signal with a resonant behavior sharp enough to accurately track the shift in transmission spectra. The third criterion pertains to the linearity of sensing which is related to the quality factor of sensors. The fourth criterion is the sensor sensitivity. If there are a limited number of data points in one frequency scan of the network analyzer, it is easier to resolve smaller shifts in the transmission spectra in response to the externally applied load when the sensitivity is higher.

The purpose of this paper is to illustrate the performance of metamaterial-based sensors. We will present in detail the principles, detecting processes and sensitivity of various metamaterial-based sensors, their differences from traditional sensors, as well as their challenges and prospects. The results show that metamaterial-based sensors possess much higher sensitivity than traditional sensors. Moreover, it is believed that metamaterial-based sensors can have potential applications in environmental sensing, homeland security and biosensing in the future.

## 2. Biosensor

Biosensors are essential in many areas, such as disease diagnostics, environmental monitoring, and food safety, and they are also vital tools in the investigation of biological phenomena. Fluorescence-based methods have proven useful in analyzing both genomic and proteomic microarrays [29,30] and in imaging, including single molecule detection inside living cells [31,32]. However, labeling molecules with fluorophores can be expensive and time-consuming. It may even be infeasible for certain applications. In addition, biological reactions generally rely on the three-dimensional structure of the biomolecules which may be affected by the addition of a fluorescent marker. Therefore, there has been a drive for bioanalytical sensing techniques that can directly detect the target molecules without labeling.

Recently, biosensing technologies based on metamaterials have attracted significant attentions from the microwave to optical frequency because of their cost-efficient and label-free biomolecule detection. According to operating frequency of sensing biomolecule and component, the metamaterial-based sensors are classified into three types: microwave biosensor, terahertz biosensor and plasmonic biosensor.

### 2.1. Microwave Biosensor

Among metamaterial components, a split-ring resonator (SRR) can be used to produce a negative magnetic permeability material (NMPM) in a time-varying H-field component of perpendicularly polarized wave incident on its surface [33,34]. In particular, the key characteristic of SRRs used to synthesize an effective NMPM is a small electrical size, which can be made smaller than the signal wavelength at resonance. Hence, SRRs can be considered as quasilumped elements suitable for the miniaturization of planar microwave devices, such as filters [35–37] and antennas [38–40].

**Figure 1.**
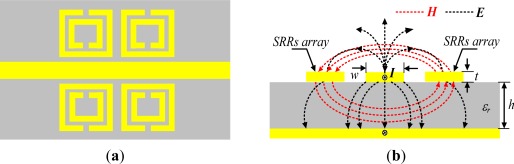
The structure of biosensing based on SRR array: (**a**) Top view of a microstrip transmission line and (**b**) Cross section of a microstrip transmission line with a pair SRRs and a schematic electromagnetic field distribution.

Recently, SRRs have been used for the fabrication of sensors and devices. For example, Lee *et al.* [41] proposed an SRR-based biosensor with a small electrical size to detect the occurrence of biomolecular binding. The structure of this biosensor consisted of two pairs of SRRs and a planar microwave transmission line, as shown in Figure 1. The planar microstrip transmission line produced a time-varying **H**-field component in a direction perpendicular to the surface of the SRRs. Moreover, the line was an open conduit for wave transmission and the electromagnetic field was not entirely confined to it. In addition, there existed a small **E**-field component along the axis of the line. Hence, the propagating mode of microwaves through the line was not a pure transverse electromagnetic (TEM) mode but a quasi-TEM mode, as shown in Figure 1(b). According to Faraday’s law, when a time-varying **H**-field component was perpendicular incident on the surface of SRR, the SRR will generate resonance. This was because the SRR can be considered to be a simple *LC* resonant circuit with a resonant frequency of *f*_0_ = 1/[2π(*LC*)]^1/2^. Based on the formula, the changes in resonant frequency depended on the changes in the inductance and/or capacitance.

**Figure 2.**
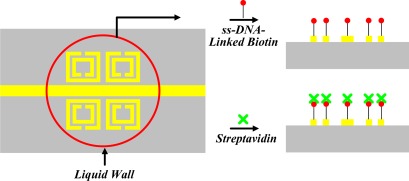
Binding bioprocess of biotin and streptavidin: the liquid wall (red circle) shows the receptacle for liquid solution confinement. The sample was immersed in biotin (red) for 12 h, rinsed, and exposed to streptavidin (green) for 6 h.

Generally, a biosensor can be defined as a device incorporating a biological sensing element related to a transducer. It consisted of the sensitive biological element, transducer or the detector element, and associated electronics or signal processors. To study the sensitivity and selectivity characteristics of SRR-based biosensors, the biosensor surface was coated with gold (Au) and the single-stranded deoxyribonucleic acid (ss-DNA)-linked biotin was used for immobilization on the Au surface because of good chemical attraction between ss-DNA and Au. After immobilization, binding of biotin and streptavidin was achieved by a bioprocess because the biotin-streptavidin binding is a well-known affinity, as shown in Figure 2.

In the absence of biomaterials, the resonant frequency of the SRR-based biosensor was 10.82 GHz. When biotin was introduced, the resonant frequency shifted to 10.70 GHz. The change of frequency in this case was Δ*f_B_* = 120 MHz. After the binding of biotin and streptavidin, the frequency shifted to 10.66 GHz. The change of frequency in this case was Δ*f_B-S_* = 40 MHz. These shifts in the resonant frequency stem from the change in the capacitance due to the binding of biotin and streptavidin. Thus, the SRR-based biosensor can be directly used for label-free biomolecular detection. Even though biosensors based on the resonator array have exhibited a remarkable resonant frequency shift, the occupied areas of the devices were relatively large and their biomolecular concentration was relatively high (∼1 μg/mL).

In the case of biosensors based on the resonator array, Lee *et al.* [42] proposed a single planar double split-ring resonator (DSRR) for biomolecular detection at microwave frequencies. The compact resonator was excited by time-varying magnetic fields generated from the 50 Ω microstrip transmission line. Similar to the SRR-based biosensor, in the absence of biomolecules, the resonant frequency of the DSRR-based biosensor was 12.35 GHz. When the ss-DNA was immobilized onto an Au surface, the frequency was shifted to 12.33 GHz. The change of frequency in this case was Δ*f*_ss-DNA_ = 20 MHz. After the binding of the ss-DNA and complementary-DNA (c-DNA), *i.e.*, DNA hybridization, the resonant frequency was further shifted down to 12.27 GHz. The change of frequency in this case was Δ*f*_hybridization_ = 60 MHz. This shift was attributed to an increase in capacitance as well as changes in inductance of the resonator surface caused by two nanometer-sized biomolecules formed by the coupling of the two biomolecules, namely, $\text{ss-DNA}(\varepsilon_{r1}^{\text{eff}})$ and $\text{c-DNA}(\varepsilon_{r2}^{\text{eff}})$, having different effective permittivities. Thus, the miniaturized microwave resonator can be utilized to detect nanosized biomolecules. In summary, the mechanism of such biosensor mainly depends on the change in the resonant frequency due to the binding of two different biomolecules onto *LC* resonators, when the resonator is excited by time-varying magnetic fields of microstrip transmission line.

### 2.2. Terahertz Biosensor

In the past two decades, terahertz waves, located in the frequency region between the microwaves and the infrared, have demonstrated attractive potential for sensing chemical and biochemical compounds [43]. Sensing the complex dielectric properties of a sample in the terahertz frequency range can directly identify the chemical or biochemical molecular composition either by detecting the resonant absorption of molecular or phonon resonances for small molecular compounds [44,45]. For large biomolecules, as no sharp absorption features are observed, dielectric changes associated with the binding of biomolecules are used for unambiguous detection and identification of macromolecules. However, many envisaged applications of terahertz sensing systems, ranging from basic instrumentation research to relevant security applications, require detection of minute amounts of chemical and biomolecular substances. This is difficult to achieve with conventional terahertz spectroscopy systems given the huge difference between the sensing wavelength and the small nanometric scale of analyte quantity associated with typical applications. Therefore, flexible and sensitive solutions to probing the dielectric properties of minute quantities of chemical or biochemical compounds are strongly desired.

Recently, Yoshida *et al.* [46] proposed a label-free sensing method by using a thin metallic mesh in the terahertz region. This sensing method was based on the change of the transmittance of terahertz radiation through a thin metallic mesh, when a sample substance was loaded on the mesh openings. The transmittance of the thin metallic mesh does not change due to the absorption. But, dominantly because of the variation of the refractive index of the sample substances near the openings, a distinct shift of the transmission dip frequency was observed for 500 pgmm^2^ of horseradish peroxidase printed on the metallic mesh.

**Figure 3.**
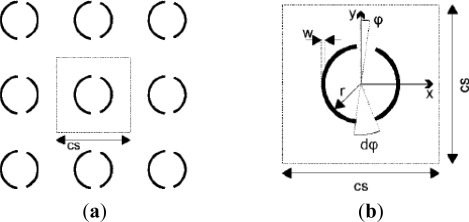
(**a**) Schematic section of aDSR based FSS adopting a square lattice and (**b**) Unit cell with radius *r* = 50 μm, width *w* = 5 μm, cell size *cs* = 220 μm, asymmetry angle *φ* = 4°, and gap angle d*φ* = 20° [48].

To achieve a higher sensitivity, the sensor needs a sharp edge in its frequency response and a point of high concentration of electric field to enable the detection of small changes in the dielectric environment [47]. Therefore, Christian *et al.* [48] obtained the high concentration point of electric field distribution by adding a second gap to split rings and breaking the symmetry. For example, a terahertz frequency selective surface (FSS) made from asymmetric split ring resonators (aDSR) of metamaterial was proposed to sense small amounts of chemical and biochemical material, as shown in Figure 3.

**Figure 4.**
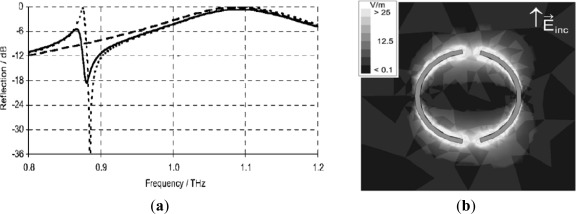
(**a**) Reflection of FSS of symmetric (dashed line) and asymmetric DSRs with *φ* = 4° for a perfect conductor (dotted line) and for gold (solid line); (**b**) The *E*-field in the resonator plane shows a strong concentration (white) at the ends of the arcs. *f* = 875 GHz, amplitude of excitation 1 V/m [48].

When the terahertz biosensor was excited with free space radiation, the reflection of the terahertz sensor showed two significant features, as shown in Figure 4(a). A broad maximum at 1,090 GHz is observed, where the length of each arc of the aDSR approximately matches half the wavelength. This dipole with antenna-like behavior shows up for the symmetric DSR (*φ* = 0°) and is not significantly affected by a small angle *φ*. By increasing the angle *φ* > 0°, the two arcs of the DSR become differently long. Around 875 GHz, the reflection of this asymmetric case showed a strong and sharp modulation of 13 dB over 13 GHz. Outside the 3 dB ranges a flank with a very high steepness of 7 dB over 4 GHz was maintained for a FSS made from gold. Moreover, it was interesting to observe that at this steep flank the electric field concentrated strongly close to the ring with amplitudes of 25 times higher than the excitation *E*_inc_, as shown in Figure 4(b). Therefore, the high sensitivity terahertz sensors were typically based on resonant structures whose frequency response was shifted by dielectric loading. Such a frequency shift can depend very sensitively on the dielectric properties of material placed in the environment of such a structure.

**Figure 5.**
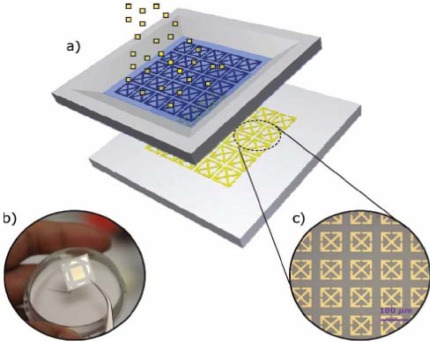
(**a**) Schematic of the micrometer-sized metamaterial resonators sprayed on paper substrates with a predefined microstencil; (**b**) Photograph of a paper-based terahertz metamaterial sample; (**c**) Optical microscopy image of one portion of an as-fabricated paper metamaterial sample [49].

Recently, Tao *et al.* [49] proposed a terahertz paper-based metamaterial (MM) device, which can be potentially utilized for quantitative analysis in biochemical sensing applications, as shown in Figure 5. Planar metallic resonators with minimum features of less than 5 μm have been fabricated on paper, using a photoresist-free shadow mask deposition technique. The fabricated paper MM devices show unique electromagnetic (EM) resonant responses at predefined frequencies, which depend on the size of resonator and could be utilized as a signature for biochemical sensing applications. In this sensing structure, the paper acted as the dielectric substrate providing both support and a material to sample and embed analytes, while the patterning metamaterial on paper substrates would offer a platform where the resonance shifts, mainly because of alterations in the SRR capacitance induced by the added analytes, can be utilized for quantitative biochemical sensing applications. Moreover, Proof-of-concept demonstrations were accomplished by monitoring the resonance shift induced by placing different concentrations of glucose solution on the paper MMs.

### 2.3. Plasmonic Biosensor

The surface plasmons are known to be extremely sensitive to the refractive index of the dielectric medium within the penetration depth of the evanescent field. This remarkable property has been used for the development of label-free plasmonic biosensors, which emerged as a leading modern technology for detection and investigation of binding events between the target analyte and its corresponding receptor on a metal surface. Currently, most of the surface plasmon resonance (SPR) biosensors use surface plasmon polaritons (SPPs). However, the SPPs can only provide an extremely small detection limit exceeding 10^−5^ refractive-index units (RIU), due to the resonant photon-SPP coupling conditions [50]. Moreover, an SPR system demands optical couplers (e.g., prisms and gratings), displays narrow operation ranges and performs over short detection distances, thus impeding its integration with low-cost, real-time and high-throughput biochips for rapid bio-analytical measurements of quantity-limited samples. Therefore, the SPP-based approach needs an improvement in sensitivity for the detection of small analytes and satisfies modern requirements of biotechnology advancing towards new nanoscale designs and promising the manipulation on the nanoscale level, size-based selectivity and selective chemical and biochemical nano-architectures [51].

Localized surface plasmons (LSPs) of metallic nanostructures seem much more suitable to match these new trends, as well as to bring new functionalities, such as spectral tenability [52,53] and strong enhancement of the local electric field [54]. But, LSP-based sensors are known to provide sensing response to refractive-index change, which is at least an order of magnitude lower than SPPs with sensitivities not exceeding 100–300 nm per RIU in spectral interrogation schemes and probe depth as small as 10 times [54], making them applicable to only a very limited number of biological species.

**Figure 6.**
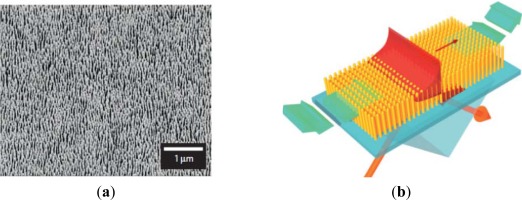
(**a**) Typical scanning electron micrograph of plasmonic nanorod metamaterial and (**b**) Schematic of the attenuated total internal reflection (ATR) measurements and flow cell [55].

In order to improve the sensitivity, a metamaterial-based plasmonic biosensor by using an array of parallel gold nanorods oriented normally to a glass substrate was proposed [55]. In this structure, the metamaterial consisted of an assembly of Au nanorods electrochemically growing into a substrate-supported, thin-film porous aluminium oxide template. The final structure represented an array of parallel nanorods occupying an area of up to 2 cm^2^, as shown in Figure 6(a). Moreover, the structural parameters can be controlled by altering the fabrication conditions with typical dimensions being in the range of rod lengths between 20 and 700 nm, rod diameter of 10–50 nm and separation from 40 to 70 nm, thus achieving a nanorod areal density of approximately 10^10^∼10^11^ cm^−2^. The lateral size and separations between the nanorods were much smaller than the wavelength of light used in the experiments, so only average values of nanorod assembly parameters were important, and individual nanorod size deviations had no influence on the optical properties that were well described by an effective medium model. For this reason, the optical properties of the nanorod arrays were very stable with respect to fabrication tolerances [56–58]. The fabrication technology made it possible to fully or partially embed the nanorods into an alumina matrix. In the Figure 6(b), the illumination of the same nanorod structure in the ATR geometry revealed the new guided mode in the near-infrared spectral range.

The metamaterial sensor worked similarly to a conventional SPP-based sensor, showing a redshift of the resonance in response to an increase in the refractive index. Furthermore, a change of the refractive index by 10^−4^ RIU caused a shift of the resonance by 3.2 nm even without any optimization of the structure. The corresponding minimum estimation of sensitivity of 32,000 nm per RIU exceeded the sensitivity of localized plasmon-based schemes by two orders of magnitude. The sensitivity of a guided wave offered by the nanorod metamaterial also exceeded the relevant parameter for commercial SPP-based sensors using spectral interrogation. Such a gain in sensitivity was due to a combination of the higher sensitivity of the metamaterial to bulk refractive-index change and a mass accumulation effect as a result of the large surface area of the nanoporous matrix. Therefore, the origin of such a high sensitivity gain for this biosensor was twofold. In nanorod arrays, the sensed substance was incorporated between the initially bare rods, and so the waveguide mode provided a better overlap between the sensing field and the sensed substance than SPR sensors. Furthermore, the effective dielectric constant ε_eff_ of the metamaterial strongly depended on the dielectric constant of the tested medium ε_d_ as a result of the modification of the plasmon-plasmon interaction in the nanorod array, thus leading to modification of resonant conditions of the guided-mode excitation caused by the sensed analyte.

Another key advantage of the metamaterial-based sensor consisted of the essentially discontinuous porous nanotexture of the nanorod matrix. This enabled the implementation of new sensing geometries and strategies, not feasible with conventional film-based SPR. Indeed, by functionalizing the nanorods and immobilizing a receptor on their surface, one can follow the binding of a selective analyte with the receptor inside the nanorod matrix. The considerably increased surface area given by the nanoporous texture of the metamaterial significantly increased the amount of biomaterial that can be incorporated into the matrix within the probe depth available, maximizing the biological sensitivity of the system. Furthermore, the distance between the nanorods can be selected to match the size of biological species of interest, giving access to a further size selectivity option that was important for many tasks in the detection of immunoassays, virus and protein.

In summary, the three biosensors have their respective characteristics, advantages and drawbacks, and the detailed comparative results were shown in Table 1.

## 3. Thin-Film Sensor

Thin-film sensing utilizing interaction between electromagnetic waves and unidentified thin-film sample substance can provide important information for many applications throughout chemistry and biology [59,60]. For example, the FSS consisting of periodic two-dimensional arrays of identical resonators, have arisen as candidates for highly sensitive chemical or biological thin film detection because it can be small and show a resonant frequency response that is tunable by design [61]. However, these ideas mainly capitalized the structure of split-ring resonators, whose natural oscillation frequencies depended critically on the permittivity of the boarding dielectrics [62]. To achieve efficient thin-film sensing, some thin-film sensors based on metamaterials are proposed and fabricated. According to operating frequency of thin-film sensing, the metamaterial-based thin-film sensors are classified into three parts: microwave thin-film sensor, terahertz thin-film sensor and plasmonic thin-film sensor.

### 3.1. Microwave Thin-Film Sensor

Despite the availability of resonators with weak freespace coupling, thin-film sensing with FSS devices is still a challenge because the sample substance has to be applied either to a specific portion of each resonator in the array or to the complete array area. Especially, when only small amounts of the substances are available, depositing the sample substances at several places introduces a high degree of inaccuracy and covering the whole area is not practical in most cases. In addition, the field confinement of the conventional thin film sensor based on circular asymmetric double split resonator (aDSR) metamaterial is relatively weak, limiting its volumetric sensitivity.

In order to sense minute amounts of sample substances, thin-film sensors have to feature a sharp transition in their frequency response. Moreover, the electric field must be confined to the portion of the sensor on which the sample substance is deposited. Based on such principles, the tip-shaped SRR metamaterial was proposed as thin-film sensor to reduce device size and resonance frequency as well as to improve the Q-factor [63]. In contrast to the traditional structures, the tip-shaped design exhibited a miniaturization and sharper dip on resonance in their transmission spectra. Furthermore, the proposed sensor can deliver the sensitivity level of 16.2 MHz/μm and the error less than a 2 μm nonlinearity when the uniform benezocyclobutene films with thickness between 100 nm and 50 μm were coated onto the fixed structure.

**Figure 7.**
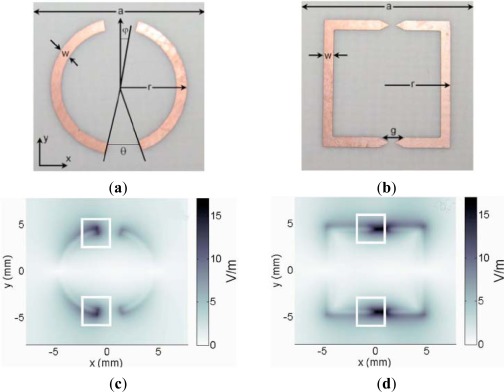
Layout of (**a**) the circular aDSR and (**b**) the rectangular aDSR with field confining tips, spatial field distribution in case of an excited field strength of 1 V/m for (**c**) the circular aDSR and (**d**) the rectangular aDSR with field confining tips [64].

To further improve the electric field distribution, a rectangular tip-shaped aDSRs with sharp tips was proposed based on the above ideas, which can offer a very high sensitivity at miniaturized scale [64]. Figure 7 showed the schematic layout and electric distribution of the unit cells for both circular and rectangular resonators, compared with the traditional structure. In the case of the circular aDSR, the strongest field amplitude was located at the end pieces of the longer resonator arm with peak values of 13.7 V/m, while the field components inside the gap remained relatively small. In contrast to that behavior, the rectangular aDSR with tips strongly confined the field into the gap with peak values of 17.1 V/m so that this area became very sensitive to changes in the dielectric environment. In summary, the rectangular design offered a miniaturization compared with circular structures. Furthermore, the tips at the end of the resonator arms concentrated the field components into a small area, increasing the volumetric sensitivity of the device. For example, the circular aDSR featured a resonant shift of 9, 24, and 48 MHz for the single covered square, the two covered squares, and the full coverage, respectively. This was considerably less than the values observed for the rectangular resonator with tips, where the corresponding shifts were 18, 36, and 78 MHz. Furthermore, the resonant frequency of the rectangular aDSR without any overlayer lied at 5.993 GHz, which was roughly 77% of the design frequency of the circular aDSR, located at 7.716 GHz. As both devices shared the same unit cell dimensions, a miniaturization by 23% was achieved.

To further improve the sensitivity of thin film sensor, a nested SRR metamaterial-based microwave thin-film sensor which combined multiple SRRs in a compactly nested structure on a single chip was proposed [65]. Its advantage was the ability to obtain sharper and deeper dips in their transmission resonance, a lower operating resonant frequency per unit area, and more regions of high electric fields, compared with traditional SRRs previously used [64]. This made the nested SRR structure very well suited for thin-film sensing applications at the microwave region. Furthermore, the nested SRR structure can achieve a higher resonant frequency shift, a higher sensitivity and a better linearity, compared with the traditional SRR structure. For example, an improved sensitivity of 28 MHz/μm or 0.41 /μm was obtained by the nested SRR metamaterial-based thin-film sensor, while the traditional SRR metamaterial-based thin-film sensor demonstrated a sensitivity of 20.68 MHz/μm or 0.21 /μm. In addition, the reduced nonlinear error of less than 182 nm (less than 7%) in the nested SRR metamaterial-based thin-film sensor was obtained, compared with the nonlinear error less than 860 nm (72.6%) of the traditional SRR metamaterial-based thin-film sensor data. Moreover, the miniaturization by 22.6% was achieved by the proposed nested SRR structure, compared with the results of the traditional SRR structure.

**Figure 8.**
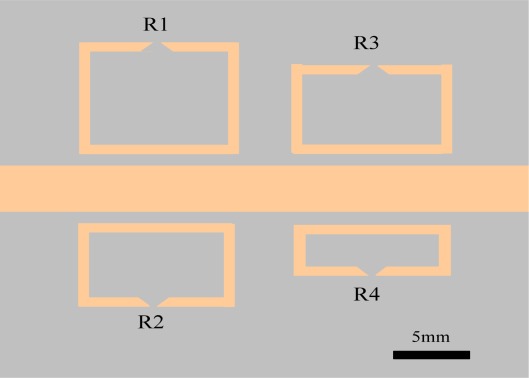
Diagram of four-channel sensor containing four SRR’s structure.

Recently, Withayachumnankul *et al.* proposed the metamaterial-based multichannel thin-film sensor. The multichannel thin-film sensor was implemented by a set of microstrip-coupled split-ring resonators (SRRs) with different dimensions, as shown in Figure 8. Each SRR exhibited a unique high-Q resonance that was sensitive to the presence of a sample in a particular area. Hence, this SRR-based sensor can function: (1) to detect different samples simultaneously to increase the throughput or (2) to characterize nominally identical samples at multiple frequencies to increase the sensor selectivity. Owing to the optimized design, sensing a low-permittivity film with a thickness as small as one thousandth of the operating wavelength was achievable.

### 3.2. Terahertz Thin-Film Sensor

Recently, the continued quest for new chemical and biological thin-film sensing modalities that improve sensitivity and take advantage of new chemical signatures has fueled a recent interest in terahertz (THz) sensing [67–69]. This is mainly due to the unique properties many materials exhibit in the THz regime. Of particular interests are those materials that respond resonantly at THz frequencies, making them more amenable to sensing in small quantities. Some examples include explosives [70,71] and DNA [72]. Detection techniques for sensing very small quantities at THz frequencies have also matured, in parallel with increasing knowledge of THz materials properties. For example, waveguide sensors have proven useful for sensing thin films of water [73] by increasing the effective interaction length. In other examples [74–77] THz micro-resonators and filters were studied to sense analytes by the frequency shift induced on the device’s resonant response. This method is reported to have increased sensitivity to the binding state of DNA samples by ∼10^3^ times over conventional free-space time-domain spectroscopy [78].

To further improve sensitivity, metamaterials have arisen as candidates for highly sensitive chemical or biological thin film detection since they can be small and show a resonant frequency response that is tunable by design. For example, small quantities of silicon (<1 ng), deposited as a film or overlayer on a planar THz metamaterial, can shift the resonant frequency by an easily measurable amount [79]. Similarly, simulations of asymmetric split-ring resonators (SRRs) indicate a possible scenario in which films as thin as 10 nm may be measured [61]. O’Hara group’s work has also shown good consistency with the principle that the resonant frequency of SRRs shifted from 0.80 THz to 0.51 THz by changing the substrate from fused silica to silicon [80].

**Figure 9.**
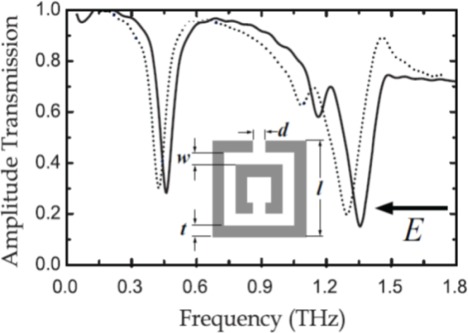
Frequency-dependent amplitude transmission of a double SRR metamaterial without (solid curves) and with (dotted curves) photoresist overlayers of 16 μm thickness [81].

To investigate the behavior of dielectric overlayers on metamaterials, the terahertz metamaterial-based thin-film sensors were fabricated by conventional photolithography techniques and consisted of square arrays of double SRRs, made from aluminum with 200 nm thickness on silicon substrates with 0.64 mm thickness [81]. Uniform dielectric overlayers from 100 nm to 16 μm thick were deposited onto fixed SRR arrays in order to shift the resonant frequency of the electric response. The metamaterial-based thin-film sensors were characterized in transmission by terahertz time-domain spectroscopy (THz-TDS) in a broadband, photoconductive switch based on system that consisted of four parabolic mirrors in a 8-F confocal geometry [82]. The measurement results are shown in Figure 9. Three distinct resonances are observed as transmission dips in the uncoated metamaterial. They are the LC resonance at *ω_LC_*/2π = 0.460 THz, the electric dipole resonance at *ω_d_*/2π = 1.356 THz, and a weaker resonance at *ω_i_*/2π = 1.160 THz due to excitation of the smaller, inner SRR. As shown by the dotted curve in Figure 9, the presence of the 16 μm thick overlayer (having a relative permittivity *ε_r_* = 2.7 ± 0.2 at 1.0 THz) causes the LC, dipole, and inner SRR resonances all to shift to lower frequencies by 36 and 60 and 78 GHz, respectively. In terms of practical sensing, however, this technique has sensing limitations inherent in these metamaterial-based thin-film sensors.

### 3.3. Plasmonic Thin-Film Sensor

**Figure 10.**
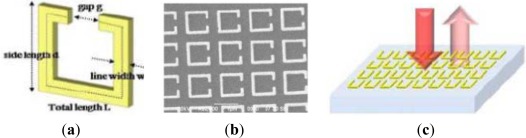
(**a**) The designed SRR unit cell; (**b**) SEM images of fabricated planar SRRs; (**c**) Schematic reflectance measurement upon the SRR-based plasmonic sensor. Here no optical coupler is required to excite plasmonic resonance. The details of the measured geometric parameters of five samples can be found in supporting information [83].

To overcome sensing limitations, a plasmonic biosensor based on split-ring resonator (SRR) array metamaterial as shown in Figure 10 was proposed [83]. The plasmonic biosensor can not only substantially ease the aforementioned burdens (coupler free, tunable operation frequencies and longer detection length), but also preserve the merits of the conventional SPR technique (excellent sensitivity, label free, quick and real-time diagnose). In addition, the SRR structures can also exhibit multiple reflectance peaks so that the SRRs can be readily employed as biosensors, especially for real-time, label-free and cell-level bimolecular thin-film detections by monitoring the shifts of reflectance peaks because of analytes binding to molecular receptors immobilized on the SRR surface [79].

When applying thin dielectric layers with different thicknesses on the SRR array, the thin-film sensor showed the distinct sensing behaviors of each resonance mode in the multi-resonance reflectance spectra. The lower-order modes possessed greater sensitivity associated with stronger localized electromagnetic field leading to shorter detection lengths within five hundreds nanometers, while the higher-order modes presented mediate sensitivity with micron-scale detection lengths to allow intracellular bio-events detection. As a result, the lower modes can be utilized to detect small targets and macro molecules including antibody-antigen interactions and the molecular recognition on the cell membrane, to gain the advantage of the excellent sensitivity and also to reduce noise from the dielectric environment, while the higher modes are facilitated to explore intracellular bio-events in live organelles and cells, due to their farther detection lengths in micron scale as well as label-free manner. Therefore, such multi-functional plasmonic biosensor can be readily employed to analyze activation-dependent cellular interactions and even a potential label-free bio-imaging device that other label-free techniques have not been achieved.

## 4. Wireless Strain Sensor

Recently, the ability to telemetrically measure strain is important to many aspects in daily life. For example, in civil engineering, remotely measuring the strength of materials (e.g., concrete) in real time, will help us to understand their transient structural behavior better (e.g., before and after an earthquake). Real-time measurement of the flexural rigidity of aircraft components during service in avionics is also an important application of telemetric strain sensing. Especially, the unrealized and critical application field of strain sensor is human medicine, where an important clinical issue is objectively monitoring the healing processes of fractured long bones [84]. However, such tasks bring about great challenges to many sectors. To date, *in vivo*, real-time monitoring of the healing process via monitoring the hardware-to-tissue load transfer has been impossible due to a lack of advanced technology. To address this problem, a bioimplantable wireless sensor system capable of monitoring the change in loading of an implantable plate should be introduced to determine the quality of the healing process. By using such a remote sensor, it is expected that a continuous healing profile of an individual patient can be recorded in daily activities.

In the last decade years, although biosensors have been studied by various groups for a wide range of applications, there exists limited data with respect to implantable microelectromechanical systems (MEMS) biosensors due to various challenges [85]. One of the drawbacks of current wireless sensors is production of a low quality factor (*Q*-factor), which can be described as the ratio of the stored to lost energy. For example, to monitor physiological parameters using telemetry-based implantable sensing systems, implantable bio-MEMS based capacitive pressure sensors can only achieve *Q*-factors of approximately 10 [86,87]. Therefore, an important requirement of these sensors is that they maintain a full on-chip resonator with a high transmission dip at resonance for telemetric sensing applications. While, reducing the size of a sensor is another major issue because of the limited space for *in vivo* implantation.

To improve the *Q*-factors, Chen *et al*. investigated symmetric and asymmetric couplings within a pair of split-ring resonators (SRRs) metamaterial. By comparing the asymmetrically coupled SRRs with the symmetrically coupled ones, it is evident to observe an additional transmittance peak with an enhanced quality factor rather than the original transmittance dip with a low quality factor [88]. Singh *et al*. analyzed the inter-SRR distance and its number density in a fixed area and found that tailoring the periodicity *Pc* of metamaterials can control the *Q* factor and the strength of the inductive capacitive (LC) resonance of SRRs [89]. Moreover, the fundamental *LC* resonance has its highest quality factor for a critical period *Pc* = λ*n*, with λ being the *LC* resonance wavelength and *n* being the refractive index of the substrate. In addition, Singh *et al*. investigated the behavior of terahertz planar metamaterials at room and cryogenic temperatures and found that thin film metamaterials showed a 14% increase in the quality factor of the *LC* resonance at liquid nitrogen temperature [90]. Melik *et al.* implemented the on-chip resonators operating at 15GHz with a *Q*-factor of 93.81 and a small chip size of 195 μm × 195 μm [91]. They effectively utilized a spiral coil geometry and cavity resonators concept, which provided a reduced area and practical implementation with a high *Q*-factor. These results showed that the use of metamaterials can improve the *Q* factor by different designs.

**Figure 11.**
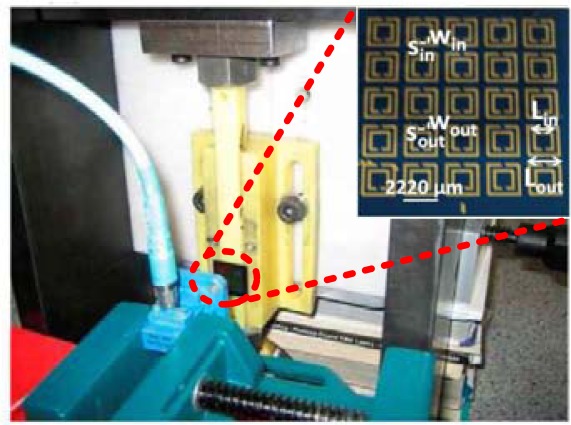
Fabricated 5 × 5 SRR array-based strain sensor under test in the compression apparatus [92].

Based on the previous studies, Melik *et al.* proposed the metamaterial-based wireless radio frequency (RF) microelectromechanical systems (MEMS) strain sensors that were highly sensitive to mechanical loading, as shown in Figure 11 [92]. The operating principle of these sensors relied on telemetrically monitoring shifts in their resonance frequencies, which were a function of the strain imparted to the associated circuit in response to externally applied loads. In operation, a cast polyamide stick was employed as the test material. When the apparatus applied compressive loads to the cast polyamide stick from 0 to 300 kgf, the sensor was mechanically deformed under stress, resulting in the operating resonance frequency shift. For example, in compression, the dielectric area and capacitance (dielectric capacitance) were decreased, the space between the metals was increased, and the capacitance between metals was decreased. These changes resulted in an overall increase in the resonance frequency. Therefore, in 5 × 5 SRR architecture, the wireless sensors yield high sensitivity (109 kHz/kgf, or 5.148 kHz/microstrain) with low nonlinear error (<200 microstrain).

To further reduce the operating frequency and to improve sensitivity, Demir *et al.* proposed nested metamaterial-based strain sensors that incorporated multiple SRRs in a compact nested architecture on a single chip to significantly achieve enhanced sensitivity in telemetric sensing [93]. This architecture introduced to implant sensing was substantially characterized by more gaps, compared with the structure of conventional SRR. This decreased the operating resonance frequency of the nested SRR sensor, compared with the classical SRR sensor. Moreover, when the external load was applied, the capacitance of the nested SRR sensor changed to a greater degree than the classical SRR, resulting in larger shifts in the transmission spectrum. For example, the unloaded operating frequency of nested SRR (506.2 MHz) was decreased relative to the classical SRR (529.8 MHz) in the free-space experiments. Further, the sensitivity of the nested SRR (1.09 kHz/kgf) was increased with respect to the classical SRR (0.72 kHz/kgf) in the free space.

**Figure 12.**
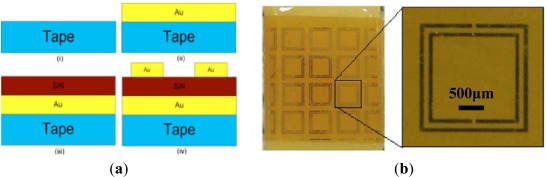
(**a**) Fabrication procedure of the tape-based flexible sensor and (**b**) The final fabricated structure of the tape-based flexible sensor [94].

Though the metamaterial-based architectures on the silicon provide the ability to achieve higher *Q*-factors and larger resonance dips in transmission, compared with conventional radio frequency (RF) structures. To enhance sensitivity and linearity, these metamaterial sensors need further mechanical flexibility. Demir *et al.* proposed flexible metamaterial-based wireless strain sensors that include arrays of split ring resonators (SRRs) to telemetrically measure strain, as shown in Figure 12 [94]. The main difference between the tape-based flexible sensors and the silicon-based sensors was the deposition of the bottom gold layer onto the vacuum tape substrate to produce a large dip at the resonance frequency. In addition, deposition of the first gold layer also guaranteed the presence of a parallel plate capacitor (between the first and final gold layers) to produce a resonance frequency shift of the tape-based flexible sensor under loading. Experimental results showed that the flexible substrate (e.g., Kapton tape) delivered greater sensitivity and a more linear response, compared with using silicon substrates.

In summary, compared with traditional RF structures (e.g., rectangular and circular coils) [91,95,96], the wireless strain sensors based on metamaterial can exhibit some advantages, including higher quality factors (Q factors) and sharper and deeper dips on resonance in their transmission. This made metamaterials very well suited for telemetric sensing applications. Moreover, metamaterial architectures can achieve higher resonance frequency shifts, leading to higher sensitivity and better linearity. In addition, by using SRR metamaterials, the operating resonance frequencies per unit area can be significantly reduced. This was especially critical for sensing applications that involve transmission through soft tissue (e.g., muscle) because such tissue strongly absorbed electromagnetic waves at very high operating frequencies.

## 5. Other Sensing

In addition, metamaterials can also be applied to other fields besides the above sensing applications. For example, Arbabi *et al.* [97] reported a highly sensitive terahertz surface wave sensor, which consisted of periodically patterned metallic metamaterials, for near field spectroscopy and sensing applications. Xia *et al.* [98] proposed a sensor based on the *ω*_m_ of a single SRR metamaterial structure with an ultra-wideband antenna, which was changed by a capacitive sensor, to wirelessly measure gas pressure, gas density, temperature, *etc*. Puentes *et al.* [99] reported a dual Mode Sensor using a metamaterial transmission line with two different operation modes for the simultaneous detection of material permittivity and position. In the first mode, the sensor was used as a resonator and the permittivity is accurately detected with a moderate cost. In the second mode, the sensor was used as a transmission line and the spatial material distribution was detected using time domain reflectometry techniques. Taya *et al.* [100] proposed an optical waveguide sensor based on metamaterial with negative permittivity and permeability. Moreover, the sensitivity of the sensor increased with the increase of the metamaterial thickness because of the surface polariton generation. Huang *et al.* [101] reported a novel Ω-shaped double negative (DNG) material-assisted microwave sensor. The sensor allowed for much higher sensitivity, compared with conventional microwave resonant sensor. Gu *et al.* [102] reported a magnetic plasmonic metamaterial which was constructed with a metal ring-shaped disk array supported by a dielectric layer on a metal film for high sensitive refractive index sensing. Xu *et al.* [103] reported a flexible metamaterial (Metaflex)-based photonic device operating in the visible-IR regime, which showed potential applications in high sensitivity strain, biological and chemical sensing.

## 6. Challenges and Prospects

In recent years, applications of metamaterials in sensing provide novel opportunities for developing a new generation of sensing technologies. Metamaterials can improve the mechanical, optical and electromagnetic properties of sensors, therefore, the metamaterial-based sensors are developing towards single molecular biosensor and high throughput sensor arrays. However, like any emerging field, they also face many challenges. For example, the performance of sensors based on metamataerials is limited by fluctuation phenomena which may be caused by various external and internal mechanisms and result in noise appearing in the sensor readout [104]. External noise sources include the quantum noise/shot noise [105], photodetector noise [106] and noise of processing circuitry, while intrinsic noise is mainly connected with the adsorption and desorption (AD) of analyte particles on the metamaterial [107]. Moreover, the AD noise remains the main limiting factor of the ultimate performance of all such devices, because each metal-dielectric structure must be exposed to some kind of environment and thus AD noise will influence to a certain degree the performance of any metamaterial structure, as well as that of conventional SPR sensors and nanoplasmonic structures.

To decrease the influence of AD noise, Djuric *et al.* [108] analyzed the effect of AD noise on the performance of microelectromechanical (MEMS) structure, and found that this noise was generated by instantaneous differences in the rates of adsorption and desorption of contaminant molecules from the resonator surface, because the analyte particles were adsorbed onto the MEMS resonator surface and/or desorbed from it, modifying its mass and causing the fluctuations of its resonant frequency. Jaksic *et al.* [109] analyzed the performance of metamaterial-based sensors which were limited by refractive index fluctuations caused by adsorption and desorption processes. To further avoid losses and scaling issues of metal-based structures, the purely dielectric configuration metamaterials instead of metal-structured metamaterials were proposed [110].

In addition, metamaterial-based biosensors still need improvements in sensitivity and accuracy, mainly because of the difficulty in obtaining high-resolution, small feature sizes on substrates where conventional photolithography techniques are difficult to apply. This is mainly due to the inability to use conventional photolithography-based microfabrication techniques where chemical solutions are generally used. Thus, the advanced fabrication technology must be developed.

In future, the fulfillment of electromagnetic response to metamaterials in the visible and infrared areas can begin a new chapter of photonics related to such novel concepts and possible applications as safety imaging, remote sensing and resonant devices. Moreover, with the arrival of micro- and nano-fabrication, new sensing possibilities are open for practical implementation of different metamaterials and the field is increasingly attracting more researches.

## Figures and Tables

**Table 1.** Detailed comparative results of three biosensor.

	
	**Microwave biosensor [42]**	**Terahertz biosensor [48]**	**Plasmonic biosensor [55]**
**Unit size**	mm	um	nm
**Operating frequency**	GHz	THz	100–800 THz
**Application field**	Detect binding of biomolecules	Detect biomolecular composition	Detect refractive index of biomolecules
**Sensitivity**	20 MHz	4 GHz	32,000 nm per RIU
**Advantages**	Low cost	High concentration of electric field	High sensitivity
**Drawbacks**	Large area	Higher cost	High cost, complicated fabricated process
